# Climatic factors driving vegetation declines in the 2005 and 2010 Amazon droughts

**DOI:** 10.1371/journal.pone.0175379

**Published:** 2017-04-20

**Authors:** Wenqian Zhao, Xiang Zhao, Tao Zhou, Donghai Wu, Bijian Tang, Hong Wei

**Affiliations:** 1 State Key Laboratory of Earth Surface Processes and Resource Ecology, Beijing Normal University, Beijing, China; 2 The State Key Laboratory of Remote Sensing Science, College of Remote Sensing Science and Engineering, Faculty of Geographical Science, Beijing Normal University, Beijing, China; 3 Joint Center for Global Change Studies (JCGCS), Beijing, China; 4 Shaanxi Jinkong Compass Information Service CO. LTD, Xian, China; 5 Beijing Engineering Research Center for Global Land Remote Sensing, Beijing, China; 6 Environmental Change and Natural Disaster, Ministry of Education, Beijing Normal University, Beijing, China; 7 College of Urban and Environmental Sciences, Peking University, Beijing, China; The Ohio State University, UNITED STATES

## Abstract

Along with global climate change, the occurrence of extreme droughts in recent years has had a serious impact on the Amazon region. Current studies on the driving factors of the 2005 and 2010 Amazon droughts has focused on the influence of precipitation, whereas the impacts of temperature and radiation have received less attention. This study aims to explore the climate-driven factors of Amazonian vegetation decline during the extreme droughts using vegetation index, precipitation, temperature and radiation datasets. First, time-lag effects of Amazonian vegetation responses to precipitation, radiation and temperature were analyzed. Then, a multiple linear regression model was established to estimate the contributions of climatic factors to vegetation greenness, from which the dominant climate-driving factors were determined. Finally, the climate-driven factors of Amazonian vegetation greenness decline during the 2005 and 2010 extreme droughts were explored. The results showed that (i) in the Amazon vegetation greenness responded to precipitation, radiation and temperature, with apparent time lags for most averaging interval periods associated with vegetation index responses of 0–4, 0–9 and 0–6 months, respectively; (ii) on average, the three climatic factors without time lags explained 27.28±21.73% (mean±1 SD) of vegetation index variation in the Amazon basin, and this value increased by 12.22% and reached 39.50±27.85% when time lags were considered; (iii) vegetation greenness in this region in non-drought years was primarily affected by precipitation and shortwave radiation, and these two factors altogether accounted for 93.47% of the total explanation; and (iv) in the common epicenter of the two droughts, pixels with a significant variation in precipitation, radiation and temperature accounted for 36.68%, 40.07% and 10.40%, respectively, of all pixels showing a significant decrease in vegetation index in 2005, and 15.69%, 2.01% and 45.25% in 2010, respectively. Overall, vegetation greenness declines during the 2005 and 2010 extreme droughts were adversely influenced by precipitation, radiation and temperature; this study provides evidence of the influence of multiple climatic factors on vegetation during the 2005 and 2010 Amazon droughts.

## 1. Introduction

The Amazon rainforest strongly influences regional and global climate dynamics and the carbon-water cycle [1, 2]. It has been estimated that the region contains one-quarter of the terrestrial species on the earth [3] and accounts for approximately one-tenth of the terrestrial ecosystem carbon stocks as well as one-tenth of global net primary productivity [4]. The region has been experiencing serious natural disasters since the 1980s [5–9]. Results simulated from a coupled regional model have projected that the extent of the Amazon rain forest may be reduced by 70% because of reductions in annual mean rainfall and a modification of the seasonal cycle [10]. Increasing tree mortality and forest fires caused by these disasters have drawn attention to the vulnerability of the Amazon’s tropical rainforests in the face of climate disturbance [9]; therefore, it is necessary to study how these disasters affect the responses of vegetation.

The Amazon region has faced frequent threat of extreme droughts since 2000, and two especially extreme droughts occurred in 2005 and 2010[11]. The 2005 drought, which was particularly severe in the southwestern Amazon, has drawn more attentions to the variation of climatic factors during the drought [5, 12–15]. However, the conclusions of these studies on the variation of solar radiation during the drought were not consistent. Samanta et al.[2010] detected the surface solar irradiance variation during the 2005 drought using shortwave radiation, photosynthetically active radiation (PAR), direct PAR and diffuse PAR, and concluded that surface radiation declined over the Amazon forests during the dry season of 2005 [13]. This conclusion conflicts with the report by Liana O. Anderson[2010] that the 2005 dry season incoming radiation was enhanced, based on visible band images derived from the GOES-12 satellite [14]. The more severe 2010 drought again put the Amazon at the center of debate [8, 16, 17]. The common features of these two droughts was that they were both associated with intense heat [12, 18] that peaked during the dry season and impacted the southwestern Amazon, although the latter affected a much larger area [16, 17]. Whereas many studies have focused on the outcomes of extreme droughts on Amazon rainforest vegetation greenness [13, 15, 19–21], the contribution of meteorological driving factors to the two droughts should be further assessed.

The two extreme droughts have provided sufficient information to analyze the relationship between climate change and vegetation greenness in the Amazon. Many studies have addressed vegetation and climate interactions during the two droughts, but some shortcomings still exist. Most of the studies focused on precipitation variation and paid little attention to temperature and radiation [8, 12, 16, 17], although the vegetation phenology in this region is also directly connected to temperature and radiation. Additionally, many studies only took into account meteorological elements that simultaneously occurred with droughts, ignoring the time-lag effects of vegetation response to climatic factors [13, 15, 17]. In recent years, a growing amount of research has claimed that vegetation responds to climate with a certain time lag [8, 22–26]. On a global scale, different vegetation types respond to the same climatic factor with different time lags, and one vegetation type also responds to different climatic factors with different patterns[22].

In this study, we used vegetation index and climate data to detect the averaging interval period of vegetation greenness response to the main climatic factors of precipitation, radiation and temperature. Based on the identified relationship between vegetation index and climatic variables, we detected how these factors contributed to the explanation of vegetation greenness and determined the major climate factor driving vegetation greenness. Finally, by synthesizing the influences of these climatic factors, we explored the relationship between vegetation index and meteorological elements in detail during the 2005 and 2010 Amazon droughts in particular detail to identify the responsible climate-driven factors of vegetation greenness variation during the droughts.

## 2. Data and methods

### 2.1 Study area

The Amazon lies the region between 10°N-20°S and 40°W-80°W. The major vegetation types of the region are evergreen broadleaf forest and savanna (S1 Fig). Because an evergreen broadleaf forest is the dominant vegetation type in the Amazon tropical rainforest [6], we categorized the Amazon rainforest as evergreen broadleaf forest. The Amazon basin in this study refers to the entire study region, excluding areas without vegetation or with altered vegetation type. Altered vegetation type refers only to pixels that have changed from one type into another type regardless of the reason. A pixel was not within the scope of our study if its type had changed. Because it is not easy to distinguish between drought and other causes of change, we did not consider specific reasons for change.

The average annual rainfall across the entire Amazon basin has been approximately 2050 mm annually since the beginning of this century[27]. The tropical rainforest that occupies a large proportion of the basin is supported by high amounts of rainfall; average annual rainfall is over 3,000 mm. There are noticeable variations in the total amounts of rainfall between the years, with the north western section receiving the maximum amounts of rainfall. The rainfall in the Amazon rainforest decreases from northwest to southeast. In the Amazon savanna area, rainfall is less and varies seasonally. The dry season of Amazon is commonly defined as consecutive monthly rainfall of less than 100 mm [6]. Dry season lengths differ greatly across the Amazon, from 0 to 5 months, roughly consistent with the distribution of annual mean rainfall [28]. Current studies on the Amazon droughts generally have considered July to September (or a few more months) to be the dry season [13, 15, 19], which is a way of simplifying the original method of consecutive monthly rainfall less than 100 mm.

Unlike precipitation, the temperature in the Amazon has no considerable seasonal or spatial variation, which is the typical situation for this region. The annual mean temperature is approximately 24–28°C and has been rising by 0.7°C since 1980, with more pronounced warming during the dry season[27]. The average temperature of the coldest month is not less than 18°C, and the absolute maximum temperature rarely exceeds 35°C. Incoming solar radiation varies seasonally because of the control of cloud cover [29], and the average incoming shortwave radiation is 212 W/m^2^, both in the rainforest and the savanna. The atmospheric conditions over the Amazon are often cloudy, especially in the wet season.

### 2.2 Vegetation index data

Vegetation index time series effectively record the seasonal and interannual variability of vegetation greenness [30]. The normalized difference vegetation index (NDVI) is currently the most widely used vegetation index and well describes the spatiotemporal distribution pattern of the vegetation canopy structure [31, 32]. Satellite remote sensing technology has become an efficient tool for environmental and vegetation monitoring because of its wide coverage and ability to be quickly acquired. AVHRR and MODIS sensors provide dynamic global vegetation observation data with 250-m to 8-km spatial resolution [30]. Long-term NDVI time-series data make the study of global vegetation dynamics possible. As reported in present studies, MODIS NDVI products from Collection 5 (C5) have substantial uncertainty resulting from large calibration degradation. However, the calibration errors were well removed in the latest MODIS Terra Collection 6 (C6) data [33, 34]. Considering that our work is related and has an extremely sensitive area, this study used MODIS NDVI products (MOD13C2) from Collection 6 (2000–2014) to characterize the greenness condition of vegetation. MOD13C2 is a monthly dataset with 0.05° spatial resolution that uses the Maximum Value Composite method to eliminate the impact of clouds, the atmosphere, and the observation angle on the data [35].

Additionally, the version 5.1 MODIS land cover type products (MCD12C1) [36, 37] for 2001–2012 were applied to distinguish Amazon vegetation types. The MCD12C1 classification product is an annual dataset with 0.05° spatial resolution. The primary goal of the MODIS land cover type products is to improve our understanding of biophysical information in regional and global models. The high accuracy of this information allows the data to be directly related to the physical characteristics of surface objects [37]. Because we aimed to investigate the climate-driven factors of certain vegetation types, it was necessary to ensure that the vegetation type did not change during the study period. We used the MODIS land cover type data to produce an unaltered vegetation type map (S1 Fig). This method was also used in a previous study on drought detection [38]. Unaltered pixels are defined as those with an unaltered vegetation type from 2001 to 2012. Removing the impact of land cover change helps to investigate the impact of drought on consistent vegetation types.

### 2.3 Climatic data

Precipitation data are the most important data for assessing drought in the Amazon [8, 12, 16, 17]. There are usually two kinds of precipitation data: CRU (Climate Research Unit) [39] and TRMM v7 (Monthly Tropical Rainfall Monitoring mission version 7) [40]. The two precipitation data types are very similar and there is not much difference between them in the Amazon region [27, 41]. Measurements from the TRMM satellite spanned an area from 50° north latitude to 50° south latitude, which covers our study area. Because the spatial resolution of TRMM precipitation data is 0.25° and the TRMM data is the most frequently used precipitation index and has been validated to perform well in detecting rainfall anomalies in Amazon drought studies [8, 14, 16, 17], it was also used in this study.

Temperature and radiation data are also important meteorological data for the analysis of drought in the Amazon. Monthly temperature data were obtained from CRU version 3.21 [39]. The datasets were derived from observations from more than 4,000 climatic stations by spatial interpolation, at 0.5° spatial resolution. Incoming shortwave solar radiation data were obtained from the version 5.2 CRU-NCEP (National Centers for Environmental Prediction) datasets, which combined CRU climate data and NCEP reanalysis data. The shortwave radiation data have the same temporal and spatial resolution as the CRU temperature data [42]. The CRU datasets have been evaluated and applied in studies of vegetation-climate interactions [22, 43–45] as well as in tropical rainforest climates [6].

To match the vegetation index data with the different spatial resolutions, we resized the climate data using the nearest neighbor resampling method. The advantages of the nearest neighbor method include simplicity and the ability to preserve original values in the unaltered scene[46].

### 2.4 Time-lag effects of vegetation response to climatic factors

In this study, we first used the Windowed Cross-Correlation (WCC) method (Boker et al. 2002), which has been frequently used [14, 22, 23], to analyze the time-lag effects of vegetation greenness response to precipitation, radiation and temperature in non-drought years throughout the Amazon region. Based on the correlation between vegetation and climatic factors, we then established a multiple linear regression model to investigate how the three climatic factors explained vegetation greenness. Finally, we assessed the change in vegetation and climate during the two extreme droughts in 2005 and 2010 to determine the fundamental climatic fluctuations that contributed to the vegetation index variations.

The WCC methods aimed to identify the most significant correlation between NDVI and each climatic factor, and the corresponding lag time. The models were as follows:

$$NDVI\left(i,j\right)=k_{pn}\left(i,j\right)*PRE\left(i,j\right)+b_{pn}\left(i,j\right)$$ (1)

$$NDVI\left(i,j\right)=k_{sn}\left(i,j\right)*SWD\left(i,j\right)+b_{sn}\left(i,j\right)$$ (2)

$$NDVI\left(i,j\right)=k_{tn}\left(i,j\right)*TMP\left(i,j\right)+b_{tn}\left(i,j\right)$$ (3)

where *k*_*pn*_, *k*_*sn*,_ and *k*_*tn*_ refer to the regression coefficients of precipitation, shortwave radiation and temperature at n months averaging interval, respectively; *NDVI* is the monthly NDVI time series from 2000 to 2012, excluding 2005 and 2010; and *PRE*, *SWD* and *TMP* refer to average precipitation, radiation and temperature time series with a lag of n months, respectively. For example, n = 0 means the immediate impact of climatic factors on NDVI as they occur, and n = 1 means a one-month lag and represents the impact of the average of the current month’s and the previous month’s climatic factors on NDVI. The analysis was based on the pixel scale, where (i, j) refers to the pixel located in the row i and column j. Based on the unchanged vegetation type map, we conducted a statistical analysis of time lags for different vegetation types in relation to different climatic factors. The determination coefficient R^2^ of the regression equation was used as an index to evaluate the correlations between NDVI and climatic factors. The highest R^2^ indicated the strongest NDVI response to a climatic factor. The time lag corresponding to the highest R^2^ was regarded as the optimum time lag.

### 2.5 Explanations of climatic factors on vegetation and the dominant climate factor

Based on the correlation models of vegetation and each climatic factor, we developed a multiple linear regression model between NDVI and three climatic factors (precipitation, shortwave radiation, and temperature) to assess their comprehensive impact on vegetation greenness, that is, an overall explanation of climatic factors on vegetation index variation. Two indices were adopted to indicate the contribution. One was the explanation (R^2^) of each climate factor on NDVI. And the other was the standardized regression coefficient of each climate factor. The details about the methods can be found in S1 Text.

### 2.6 Variations in vegetation and climatic factors

The two droughts both occurred with the arrival of a dry season (July- September) [17]; therefore, we first calculated the NDVI anomalies during the dry season to characterize the impact of the droughts on vegetation. The calculation was performed at the pixel scale using the following anomaly index[14], and an identical reference period (i.e.2000-2014, excluding 2005 and 2010) was applied:

$${\text{NDVI}}_{\text{yanomaly}}\left(i,j\right)=\frac{\text{NDVIy }\left(i,j\right)-MEAN\;\left({\text{NDVI}}_{2000-2014}\left(i,j\right)\right)}{STD\;\left({\text{NDVI}}_{2000-2014}\left(i,j\right)\right)}$$ (4)

where NDVIy_anomaly_(i, j) represents the dry-season NDVI anomaly of pixel (i, j) in 2005 (or 2010); NDVIy(i,j) represents the monthly average NDVI of pixel (i, j) in the 2005 (or 2010) dry season; *MEAN*(NDVI_2000-2014_(i,j)) represents the average dry-season NDVI from 2000 to 2014 excluding 2005 and 2010, which indicates the dry-season NDVI conditions in non-drought years; and *STD* represents the standard deviation of the dry-season NDVI in non-drought years. The anomaly value was normalized by division by the mean and indicates the amplitude of the variation relative to the mean.

We computed anomalies of the climatic factors during a period that affected dry-season (the most severe drought period) NDVI in the two droughts based on the lag effect. Consider precipitation as an example to elaborate the method(S2 Text). The calculation of shortwave radiation and temperature anomalies follow a similar approach. For a specific pixel (i, j), we first determine the time lag of NDVI response to precipitation. If the lag is 0 months, then calculate the precipitation anomalies of the dry season (July to September), then the same with that of NDVI. If the lag is 1 month, precipitation anomalies from June to September would be calculated; if the lag is two months, precipitation anomalies from May to September would be calculated, etc.

## 3. Results

### 3.1. Time-lag effects of vegetation response to climatic factors

**Fig 1:**
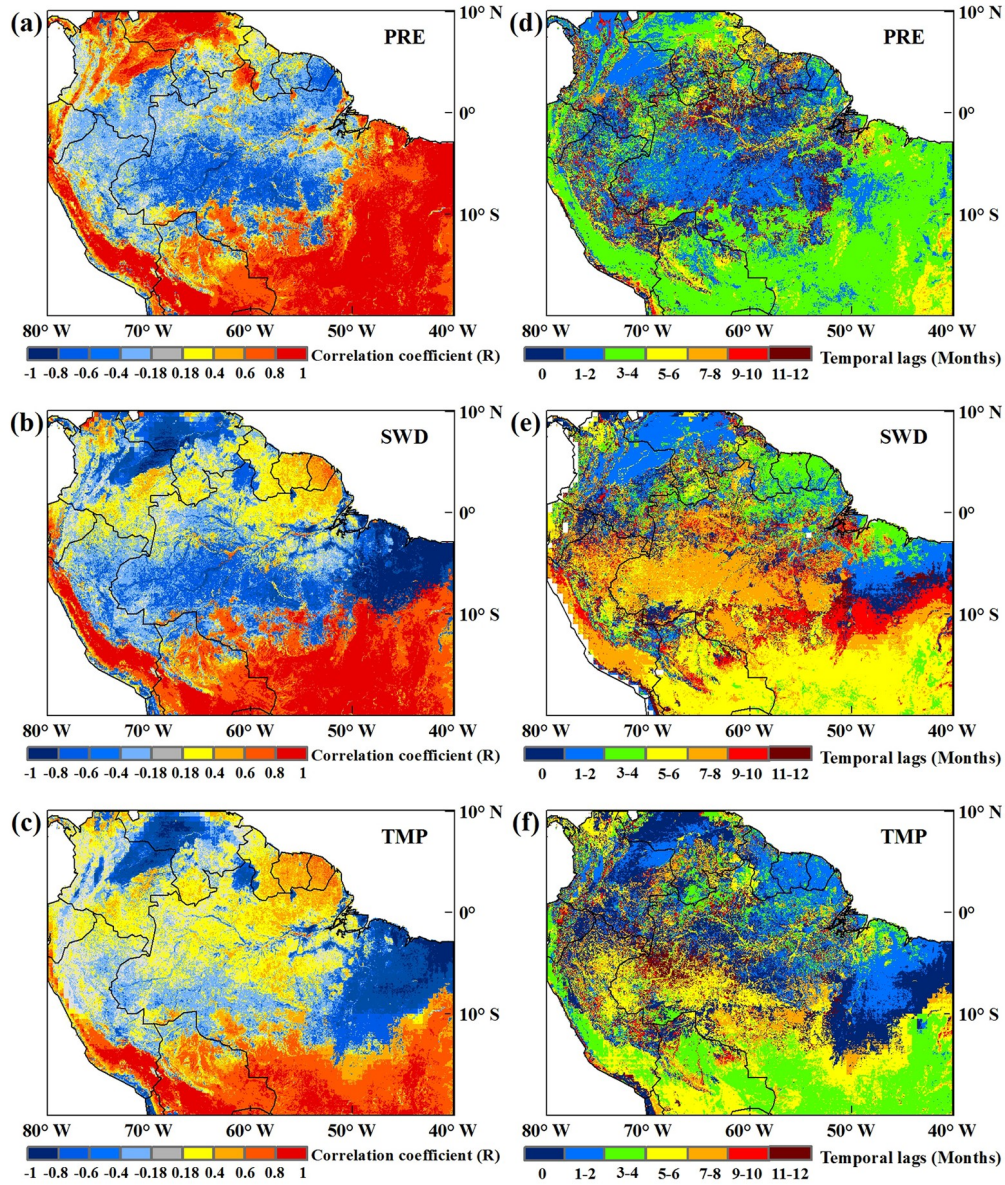
(a-c) Maximum correlation coefficient between NDVI and each climatic factor (during the period of 2000–2012 excluding 2005 and 2010); (d-f) the corresponding time lag. From top to bottom, images are for precipitation (PRE), shortwave radiation (SWD), and temperature (TMP).

Vegetation index and climate data were used in this study to detect the averaging interval period of vegetation greenness response to the main climatic factors of precipitation, radiation and temperature. The results showed that there were differences of averaging interval period among different vegetation types responding to the main climatic factors in non-drought years (Fig 1). As was previously mentioned (see 2.1), the Amazon rainforests receive much more precipitation than the savanna region. The correlation between precipitation and NDVI is negative in the rainforest (Fig 1a), which means that an increase in precipitation would inhibit the greenness of vegetation. Greening of tropical forest was negatively related to precipitation on average, which suggests that vegetation photosynthetic activity was higher during drier months than wetter months [20, 34]. However, in the savanna region, which is subject to relatively low precipitation, vegetation greenness was positively correlated with precipitation; that is, a precipitation increase would promote vegetation greenness. Therefore, vegetation photosynthesis would be higher in the wet season for the savanna in contrast to the rainforest [34]. The time lag of the vegetation greenness response to precipitation differed across the region [14]. The rainforest responded to precipitation more quickly, mainly with a time lag of 0–2 months; the savanna responded to precipitation with a time lag of 3–4 months (Fig 1d). The spatial variability of time lags across the Amazon region may be related to the availability of regional precipitation. As revealed by Donghai Wu et al.[2015], the lag time of vegetation in arid areas is longer than that in humid areas, which indicates that the demands of vegetation in arid areas for water are insufficient[22]. The drier the area, the more the vegetation greenness relies on the precipitation in the previous months, not only the current month.

The response of Amazon vegetation to shortwave radiation and temperature also varied spatially with different local water and heat conditions. As the tropical regions experience frequent cloud cover related to rainfall [34], the heat or incoming solar radiation is thus affected, and the distribution of rainfall has an influence on the response of NDVI to radiation. In the northern rainforest and savanna, the correlation between NDVI and shortwave radiation was positive (Fig 1b), which indicated that an increase in shortwave radiation could promote the greenness of vegetation. However, in the central and southwestern rainforest, the shortwave radiation had a negative impact on vegetation, with increased shortwave radiation inhibiting vegetation greenness. Additionally, the central and southwestern rainforest and savanna responded to shortwave radiation with an obvious time lag of 5–8 months (Fig 1e). Vegetation response to temperature was relatively consistent with response to shortwave radiation, in terms of the distribution of positive and negative effects. However, the correlation between temperature and NDVI was weaker when compared with shortwave radiation (Fig 1b and 1c), and temperature-related time lags in the southwest rainforest and savanna regions ranged from 3–6 months (Fig 1f).

**Fig 2:**
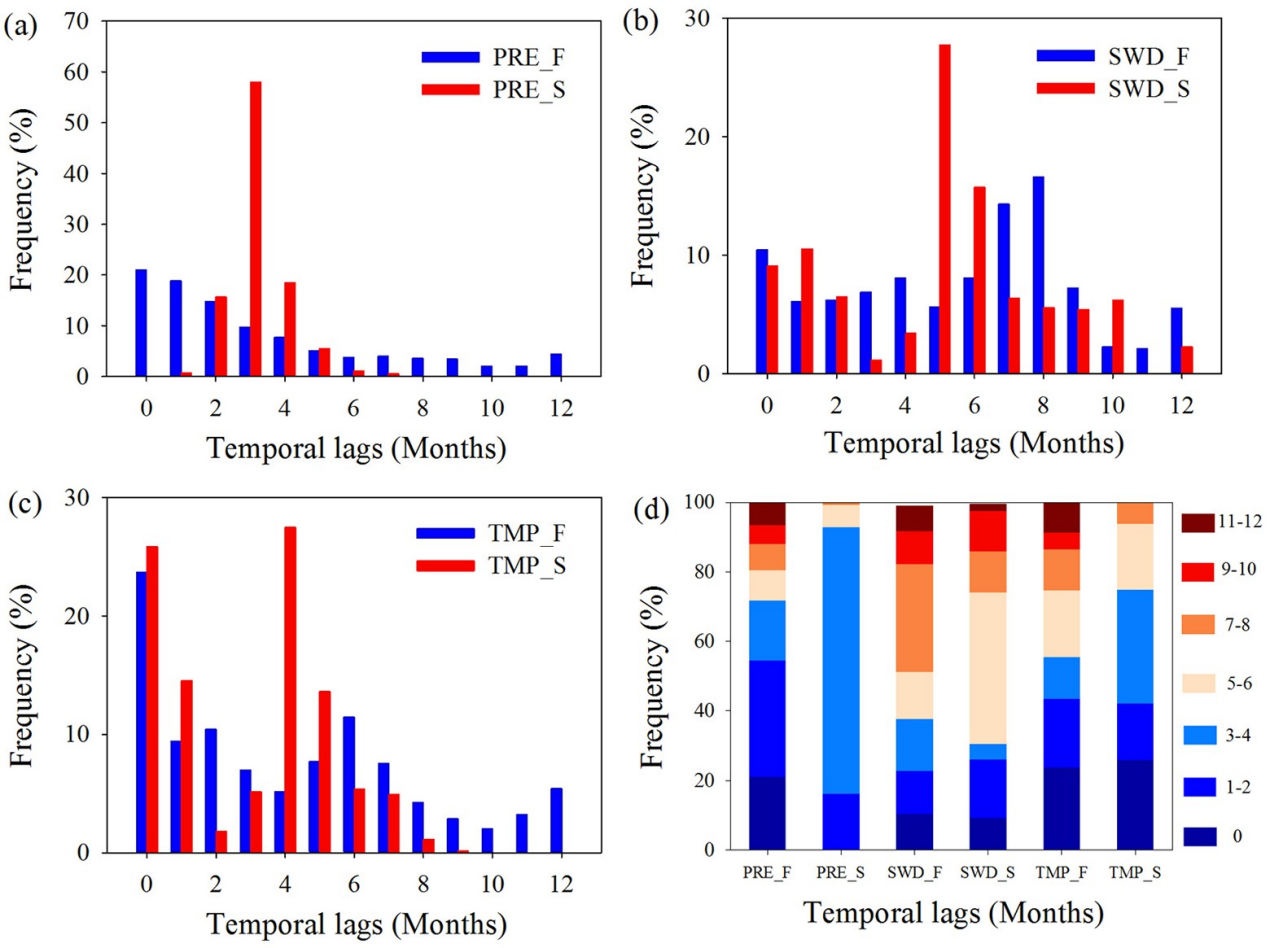
Frequency histograms of time lags for different vegetation types. Histograms represent pixel proportions at various time lags from 0–12 months for different vegetation types to precipitation (PRE) (a), shortwave radiation (SWD) (b) and temperature (TMP) (c), and a cumulative frequency map for different vegetation types combining Figs a-c (d). F refers to tropical rainforest of evergreen broadleaf forest; S refers to savanna.

The frequency histogram (Fig 2) illustrates that vegetation in the Amazon rainforest intensively responded to precipitation within a 0–2 month lag, which accounted for 54.48% of the rainforest pixels; in the savanna, the strongest responses of NDVI to precipitation occurred with 3–4 month lags, which accounted for 76.49% of the savanna pixels (Fig 2a). Rainforest vegetation greenness correlated most with shortwave radiation at 0-, 7- and 8- month lags, which accounted for 41.35% of the rainforest pixels, whereas savanna greenness responded most to shortwave radiation with 5- or 6- month lags (43.50% of pixels) (Fig 2b). For temperature, 23.68% of the rainforest pixels responded to temperature changes in the same month, whereas 81.46% of the savanna pixels correlated most with temperature at 0–1, and 4–5 month lags (Fig 2c).

The distribution of time lags shows that in most Amazon areas, vegetation greenness responded to precipitation, radiation and temperature within a certain interval period. To show that considering time lags can explain the response to climate of Amazonian vegetation, we simultaneously compared the differences between the determination coefficients R^2^ of each model with and without a time lag and calculated the regional average R^2^.

**Fig 3:**
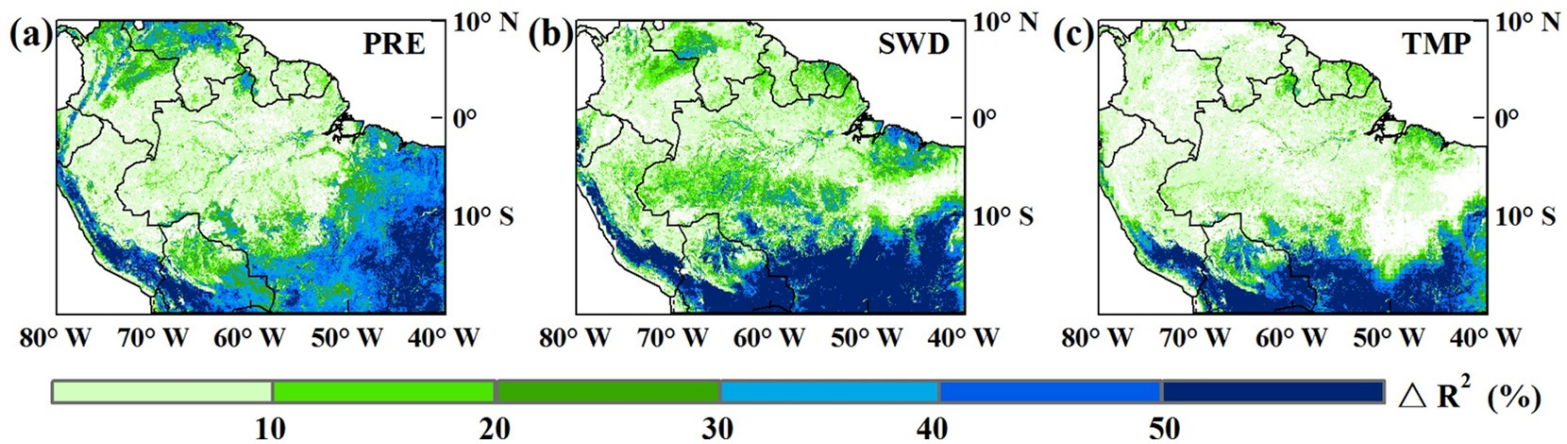
Difference between determination coefficients R^2^ for each correlation model of vegetation and climatic factors with or without time lag. (a)△R^2^ for precipitation; (b)△R^2^ for shortwave radiation; (c)△R^2^ for temperature.

From the R^2^ difference distribution map (Fig 3) and average R^2^ for the two different vegetation types (Table 1), it is evident that considering a time lag largely improved the statistical correlation between each climatic factor and NDVI to a large extent. In most areas, R^2^ obviously improved. Specifically, the average R^2^ of precipitation, radiation, and temperature increased by 5.4%, 9.7%, and 4.7%, respectively, in the tropical rainforest. Correlations for precipitation, radiation, and temperature in the savanna area increased by 41.3%, 42.1% and 25.6%, respectively.

**Table 1:** Average and standard deviation of R^2^ for Amazon rainforest, savanna and the basin (i.e., the entire study area excluding non-vegetated pixels and type-changed pixels).

		Forest	Savanna	Basin	Forest	Savanna	Basin
		Mean			SD		
PRE	No Time Lag	0.090	0.273	0.147	0.089	0.106	0.126
	0–12 Month Lag	0.144	0.686	0.324	0.113	0.113	0.278
SWD	No Time Lag	0.055	0.244	0.109	0.065	0.270	0.170
	0–12 Month Lag	0.152	0.665	0.320	0.117	0.151	0.271
TMP	No Time Lag	0.057	0.216	0.105	0.068	0.221	0.150
	0–12 Month Lag	0.104	0.472	0.232	0.092	0.172	0.223

### 3.2. Explanation of three climatic factors on NDVI and climate-driven factor

**Fig 4:**
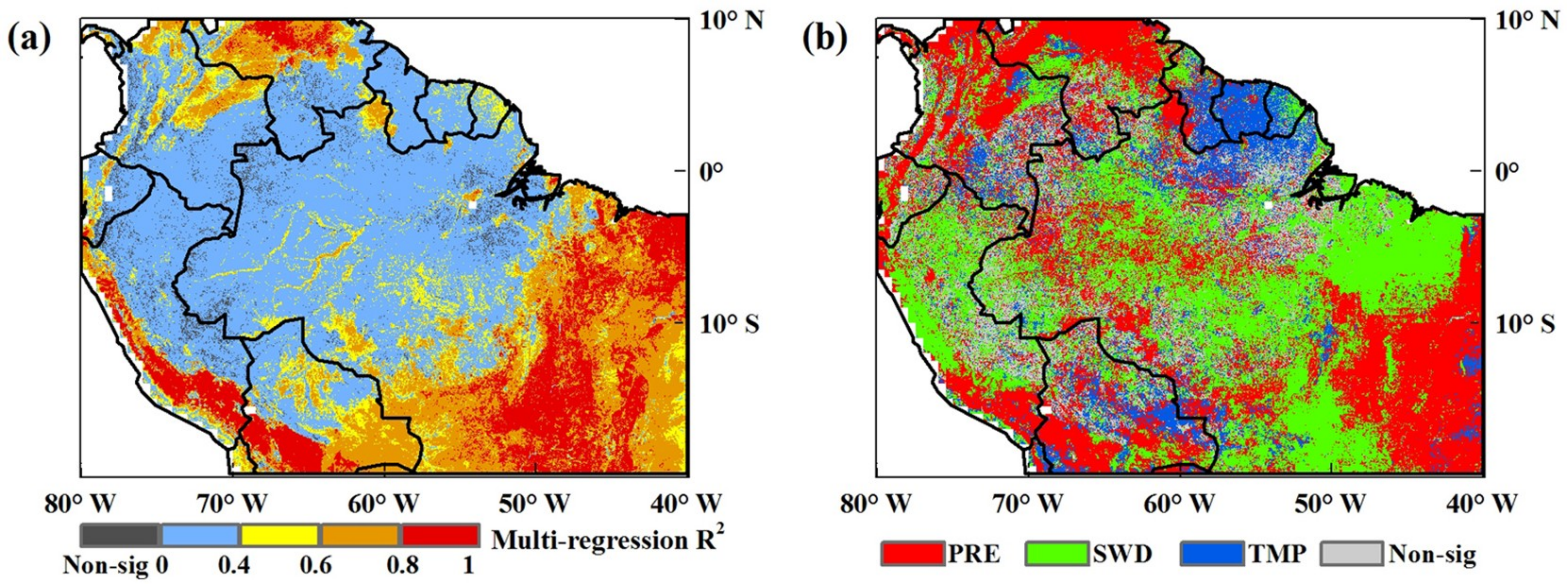
(a) The spatial distribution map of the determination coefficient (R^2^) from the multiple linear regression model of NDVI and climatic factors considering time-lag effects. (b)The spatial distribution of the main climate-driven factors related to vegetation greenness in the Amazon. Areas driven by precipitation are depicted in red, shortwave radiation in green, and temperature in blue.

A multiple linear regression model was used to estimate the overall explanation of how the three climatic factors (precipitation, shortwave radiation, and temperature) impacted NDVI as well as the relative contribution of each climate factor to NDVI. As shown in the distribution map of the determination coefficient (Fig 4a), the model passed the significance test (*α* = 0.05) for most areas of the Amazon (96.69%). Overall, the three climatic factors explained the NDVI variation in the savanna better than in the rainforest, especially the southwest rainforest. Table 2 shows the regional average explanations of variations in NDVI for two different vegetation types under two conditions: no time lag and time lag. Comparisons suggested that in the tropical rainforest, the three studied climatic factors explained 14.85% of NDVI variation on average if time-lag effects were ignored. When time-lag effects were considered, the explanation improved by 6.53%; in the savanna region, climatic factors explained 53.07% of NDVI variation when time lag was ignored. This values increased by 22.33% when time-lag effects were considered, a relative increase of 42.08%. Throughout the Amazon region, only 27.28% of vegetation index variation was explained by the three climatic factors without the time lag. When lag effects were considered, this value increased to 39.50%, a relative increase of 44.79%.

**Table 2:** Comparisons of the comprehensive explanations of the three climatic factors to vegetation greenness with or without time-lag effects in the Amazon evergreen forest, savanna and the basin (i.e., the entire study area excluding non-vegetated pixels and type-changed pixels).

	Forest	Savanna	Basin	Forest	Savanna	Basin
	Mean(%)			SD(%)		
No Time Lag	14.85	53.07	27.28	10.31	17.60	21.73
0–12 Months Lag	21.38	75.40	39.50	12.04	10.48	27.85

Table 3 shows the respective contribution of each climate factor on vegetation greenness when time-lag effects were considered. In the tropical rainforest, on regional average, the three studied climatic factors (precipitation, radiation, and temperature) explained 12.84%, 10.80% and 2.54% of NDVI variation, respectively; in the savanna region, climatic factors explained 23.21%, 38.31% and 2.19% of NDVI variation. Throughout the Amazon region, precipitation and shortwave radiation could explain 16.75% and 18.31% of the vegetation index variation, respectively; only 2.45% was explained by the temperature. These results suggest that precipitation and shortwave radiation have stronger impacts on vegetation greenness in the Amazon region, whereas temperature has less influence.

**Table 3:** Average and standard deviation of explanation (R^2^) of each climatic factor on vegetation greenness in Amazon evergreen forest, savanna and the basin (i.e. the whole study area excluding non-vegetated pixels and type-changed pixels).

	PRE	SWD	TMP	PRE	SWD	TMP
	Mean(%)			SD(%)		
Forest	12.84	10.80	2.54	18.59	18.72	12.52
Savanna	23.21	38.31	2.19	34.73	35.85	13.36
Basin	16.75	18.31	2.45	25.88	27.70	12.76

Standardized regression coefficients of the regression model in non-drought years (from 2000 to 2012, excluding 2005 and 2010) were also applied to indicate the relative contribution of precipitation, shortwave radiation and temperature to variation in NDVI and thus obtain the dominant factor of vegetation greenness. We defined the dominant driving factor as the one with the maximum absolute value of the standardized regression coefficient on the premise that it passed the significance test. By comparing the standardized regression coefficients of the three climatic factors and NDVI, we devised a spatial distribution map of climate-driven factors on vegetation change in the Amazon (Fig 4b). It is clear from the map that for most regions of the Amazon, vegetation greenness is primarily affected by precipitation and shortwave radiation, whereas temperature has less influence. In the rainforest, the pixels indicating precipitation as the climate-driven factor accounted for 24.74% of all the evergreen broadleaf forest pixels, whereas radiation and temperature accounted for 32.99% and 19.04%, respectively; in the savanna, the proportions of precipitation, shortwave radiation and temperature as climatic factors were 52.46%, 42.37%, and 4.24%, respectively, which revealed precipitation and shortwave radiation as the dominant driving forces for both vegetation types in the Amazon. The southwestern rainforest was dominated by shortwave radiation (Fig 4b). The central rainforest was dominated by precipitation and shortwave radiation. Temperature-dominated areas were only concentrated in the northeastern rainforest. The spatial patterns are comparable to previous works, which reported the global spatial distribution of the main climate-driven factors to the vegetation greenness (Sassan et al. 2013) [10].

### 3.3. Vegetation and climate variations in 2005 and 2010

**Fig 5:**
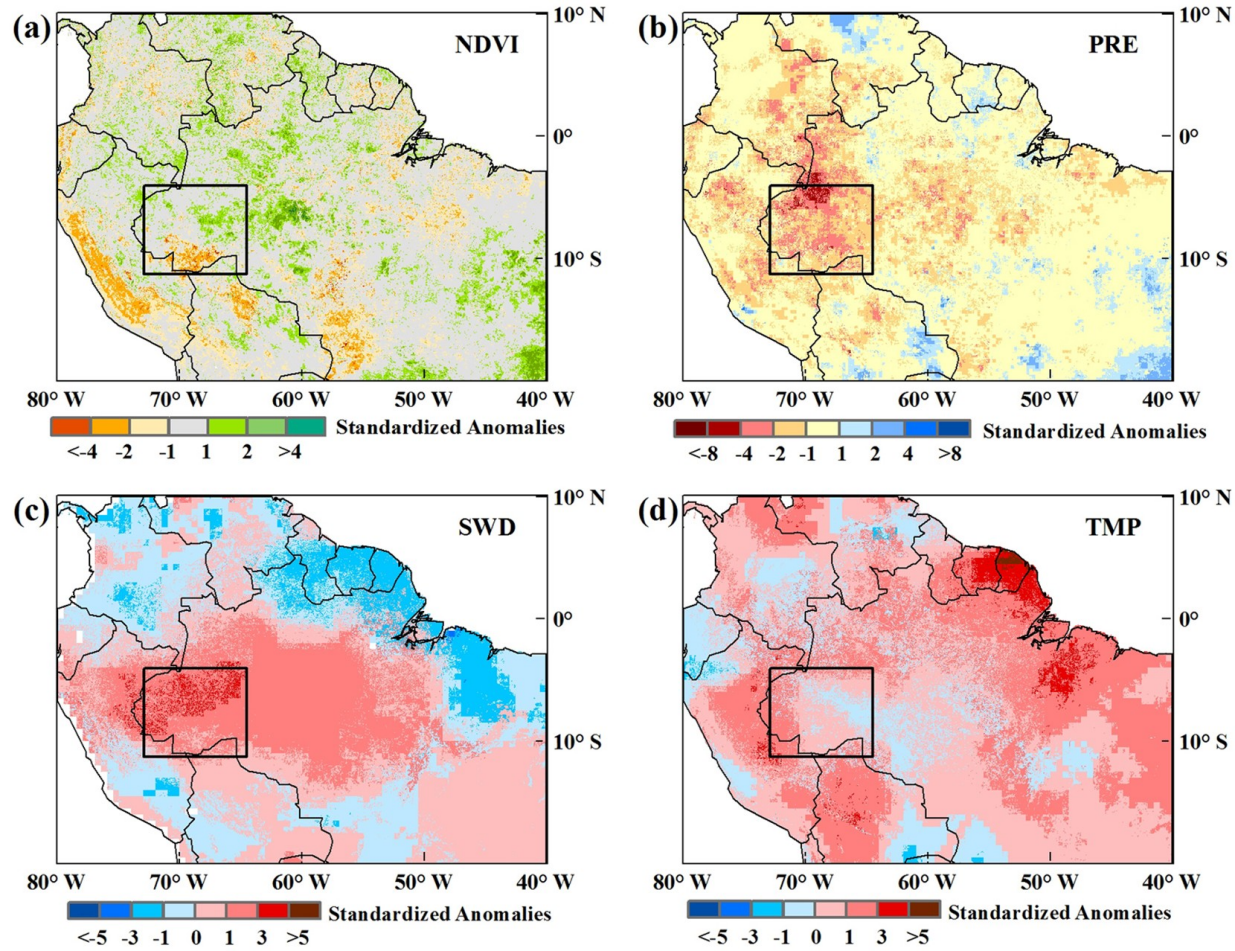
(a) Spatial distributions of dry-season NDVI anomalies in 2005; (b-d) spatial distributions of three climatic factors’ anomalies during the period with a certain month’s lag during the 2005 dry season. Normalized anomalies beyond ±1.0σ represent a significant departure from the average of non-drought years. The black rectangular area represents the common epicenter of the two extreme droughts.

**Fig 6:**
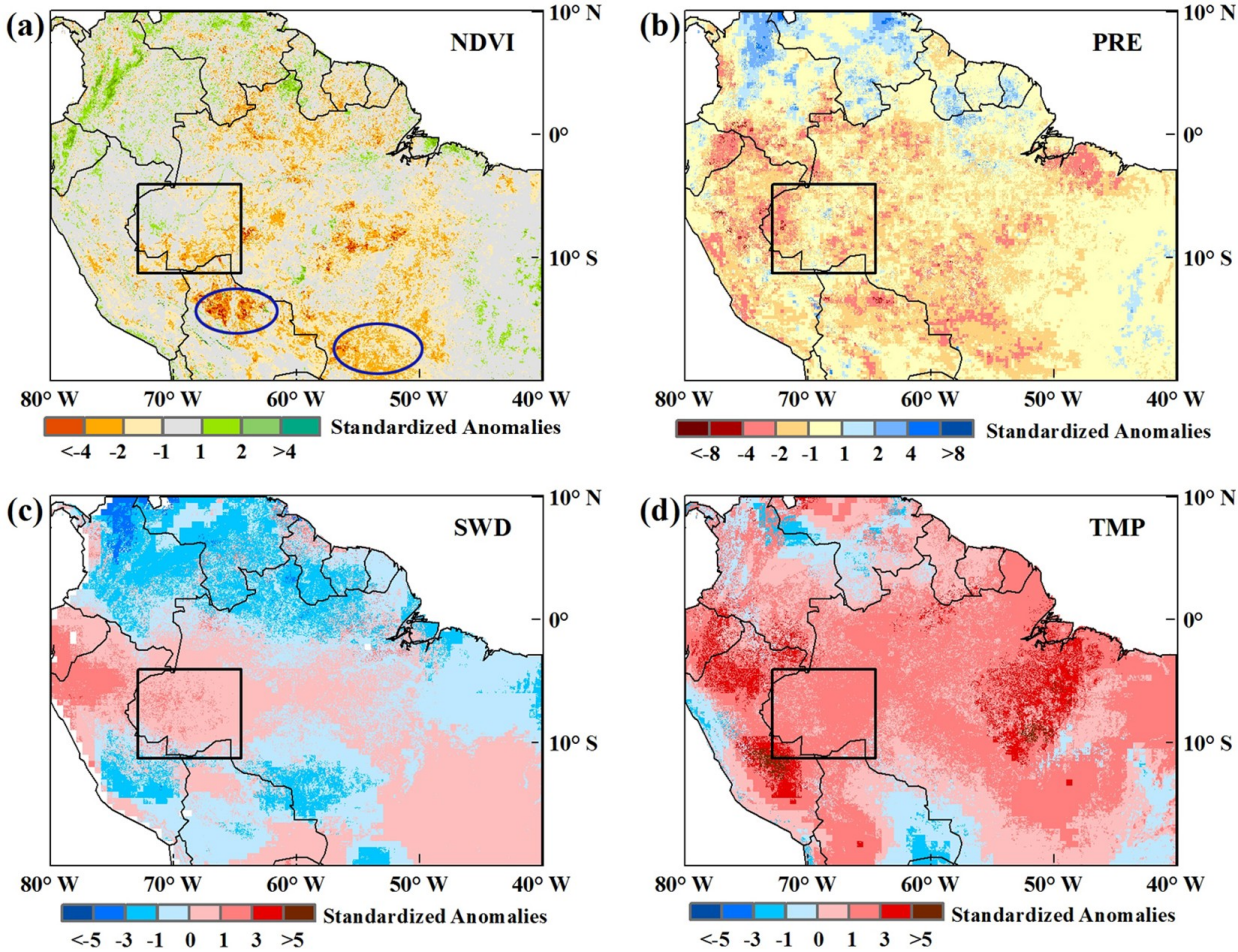
(a) Spatial distribution of dry-season NDVI anomalies in 2010; (b-d) spatial distributions of three climatic factors’ anomalies during the period with a certain month’s lag during the 2010 dry season (considering the time-lag effects). Normalized anomalies beyond ±1.0σ represent a significant departure from the average of non-drought year. The black rectangular area represents the common epicenter of the two extreme droughts.

The 2005 and 2010 Amazon droughts both occurred along with the arrival of the dry season, and we first analyzed changes in the dry-season NDVI for each year from 2000 to 2014. Dry-season NDVI anomalies (S2 Fig) showed that there were sharp reductions in the southwest rainforest and part of the savanna region in 2005. And in 2010, the NDVI showed obvious decreases on a large scale. The distributions of the 2005 and 2010 NDVI anomalies were consistent with previous findings[17]. The 2005 and 2010 Amazon droughts were both associated with vegetation decline, but the dry-season NDVIs for the two periods were characterized by declines of different magnitudes (Figs 5a and 6a). The NDVI decline in 2005 was clearly visible in the southwestern Amazon rainforest, although the scale was not large. The extent of the 2010 NDVI decline exceeded that of 2005, with significant decreases in most areas.

Regarding climatic factors, distinct variations in precipitation, radiation and temperature with specific time lags that influenced the dry-season NDVI most during the droughts could be visually observed. Anomalies of each climatic factor showed a comparable variation pattern for both the 2005 and 2010 droughts, mainly with decreasing precipitation and increasing shortwave radiation and temperature. However, the intensity and extent of the three climatic factors during the two droughts had differences. Precipitation decreased markedly compared with non-drought years, which spread over a larger area in 2010 than 2005. The patterns were highly consistent with the study of Simon L. Lewis [16]. Temperatures increased across much of the region during both droughts [18], and the rise amplitude was greater in 2010 than 2005. Shortwave radiation increased above non-drought years in the south and central rainforest and parts of the savanna regions, significantly in 2005 and weakly in 2010.

**Fig 7:**
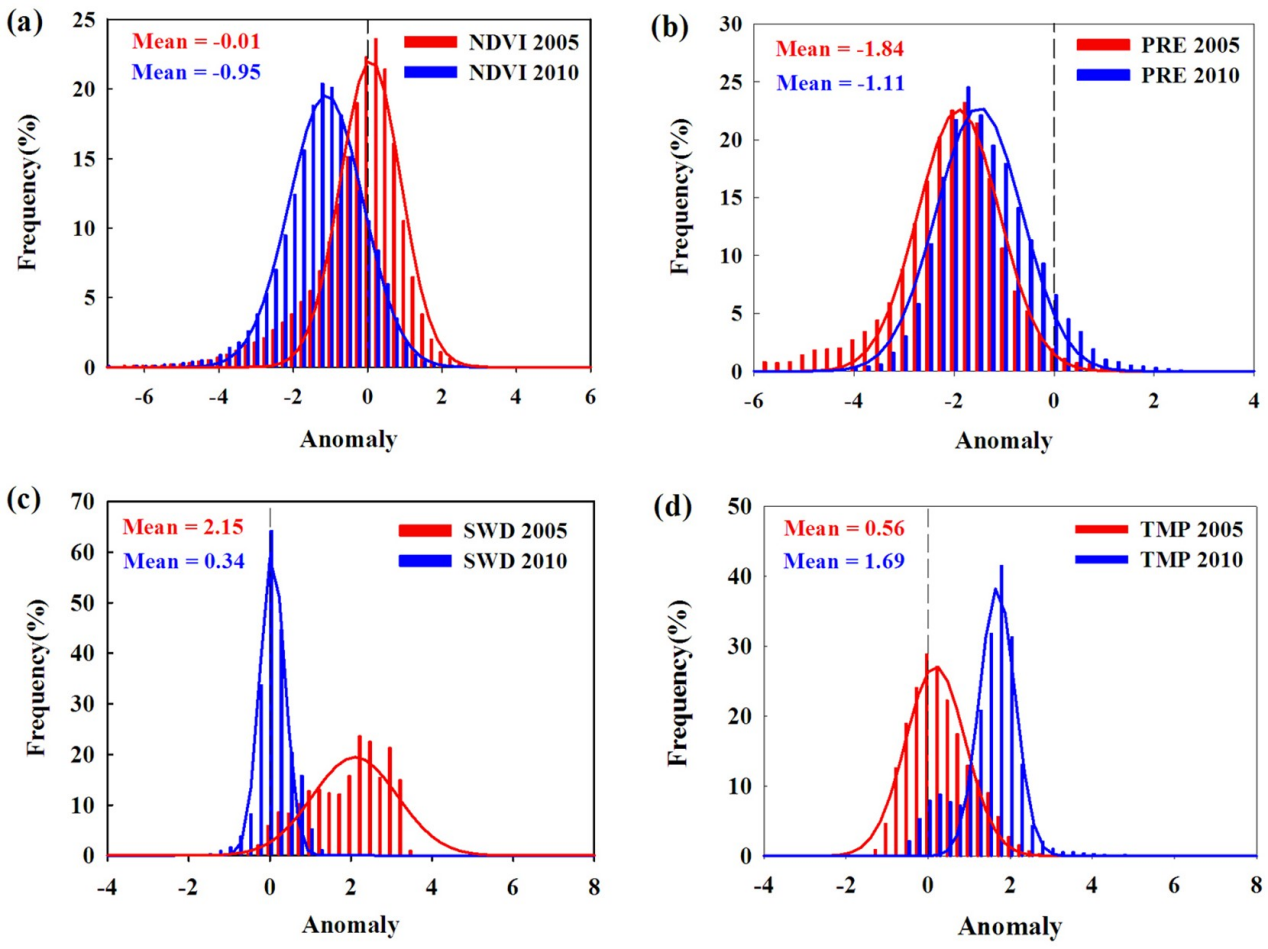
(a) Frequency distributions of NDVI anomalies at the common epicenter in 2005 and 2010; (b-d) frequency distributions of the three climatic factors’ anomalies at the epicenter in 2005 and 2010.

**Fig 8:**
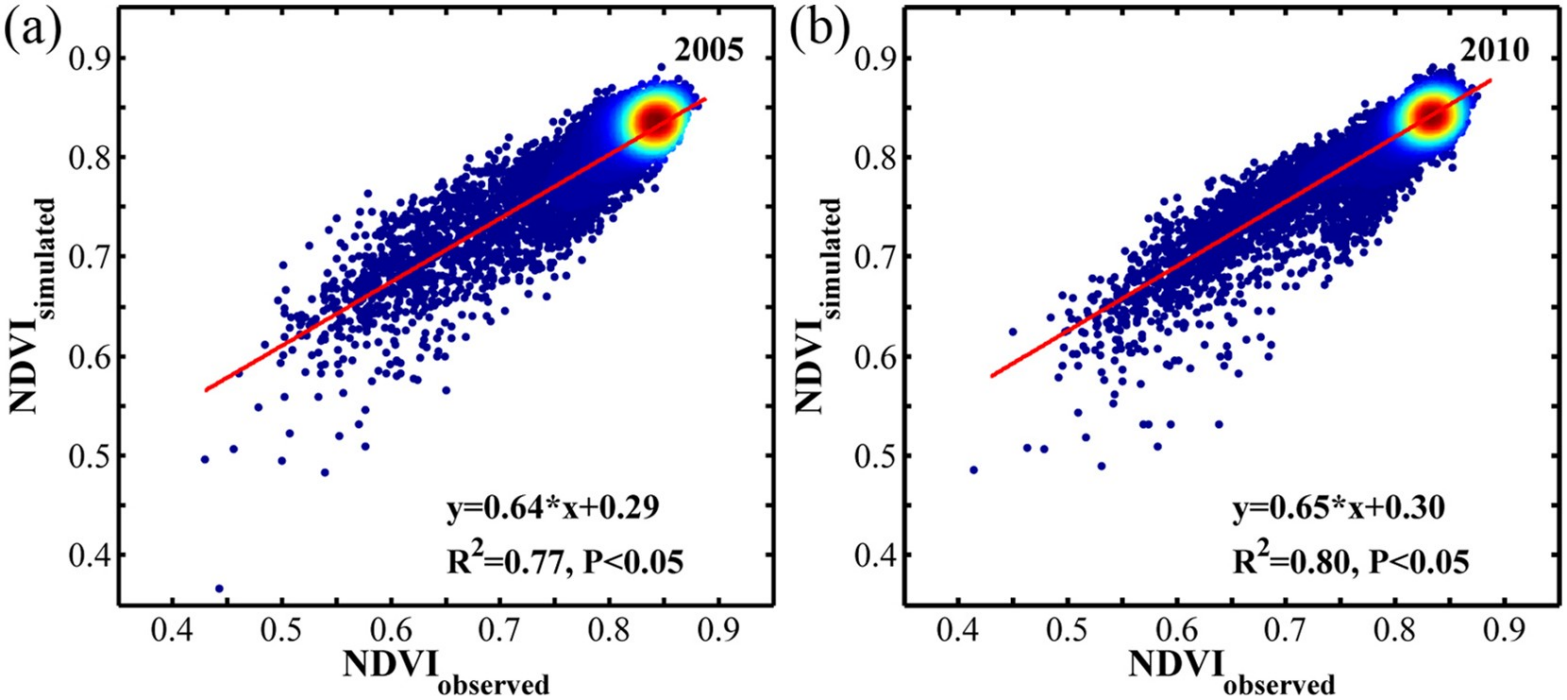
The relationship between the observed NDVI and the simulated NDVI of dry seasons in 2005 (a) and 2010 (b). The color represents for the point density; red means high density and blue means low density.

As can be seen from Fig 5, there was a more severe drought-affected area in 2005, the rectangle area in the figure. From the Fig 6, we can see that there were three epicenters of droughts in 2010, which are marked by the rectangle area and two ellipse areas. To compare the differences and similarities of the influence factors of drought between 2005 and 2010, based on the anomaly distributions of NDVI and climatic factors as well as current studies [8, 16], we selected the area (Figs 5 and 6, 73-64W, 4-11S, the black rectangular area in the anomalies spatial distribution map) as a common epicenter of the two extreme droughts for further comparative analysis.

We performed a frequency distribution analysis of anomalies in vegetation NDVI and the three climate variables in the epicenter. Features of climate and vegetation change in this region were noticeable, as illustrated in the frequency distribution graphs (Fig 7): NDVI declined during the two droughts to different extents, and it indicated that NDVI decline in 2010 was more severe than in 2005. Additionally, the increases in radiation and temperature were obviously different. The degree of radiation enhancement was more remarkable in 2005, whereas the temperature rise was more distinguished in 2010.

Because of the correlation results, we calculated the percentage of climatic factors with significant variation that accounted for declining NDVI pixels at the epicenters of the droughts. Based on the anomaly values of NDVI in the epicenter, those NDVI pixels that declined significantly and whose anomaly values are less than -1 were selected. Then, the pixels with significant variations in temperature, precipitation, and radiation were marked and counted. Based on the resulting statistics, the pixels with significant variation in precipitation accounted for 36.68% and 15.69% of the total pixels that significantly decreased in NDVI in 2005 and 2010, respectively. The proportions for radiation were 40.07% in 2005 and 2.01% in 2010 and for temperature were 10.40% in 2005 and 45.25% in 2010. The results signify that the three climatic factors may all have influenced the vegetation changes during the two droughts. Additionally, their impacts were distinguished in 2005 and 2010. It can be inferred that the impact of temperature in 2010 was greater than in 2005, whereas shortwave radiation was greater in 2005.

To verify that the regression model based on data from non-drought years was also applicable for drought years, we used the precipitation, radiation and temperature of certain months that most affected the dry season NDVI most in 2005 and 2010 to simulate the dry season NDVI (NDVI_simulated_). Then, we compared the simulated NDVI with observed NDVI (NDVI_observed_) based on bootstrapped method (Fig 8). that is, the response model based on non-drought years could be applied during drought years. Moreover, that the three climatic factors could well predict NDVI also showed that all the three factors comprehensively influenced NDVI variation. Although the spatial distribution of the climatic factor anomalies in the two droughts were not very consistent with NDVI anomalies and none of the single factors could adequately explain the NDVI decline during the two droughts (Figs 7 and 8), the response model indicated that NDVI variation was a comprehensive effect of these three factors.

## 4. Discussion

The results of this study indicated that the responses of vegetation index to precipitation, radiation and temperature had obvious averaging interval periods in the Amazon region. Specifically, the same types of vegetation exhibited apparently different time lags in response to different climatic factors, and different vegetation types responded differently to the same climatic factor. Tropical rainforest vegetation responded to precipitation over a 0- to 3- month lag. S. Sassan et al. [2013] have also found that Amazon rainforest vegetation responded to precipitation over a 1- to 3- month lag [8]. Anderson et al. [2010] detected changes in vegetation caused by Amazon droughts and explored the time-lag effects of vegetation response to shortwave radiation, precipitation and atmospheric optical depth. The results of this study proved that vegetation has a clearly different time-lag response to different influencing factors. In Anderson’s study, a time lag up to six months was considered [14]; however, our results showed that the time lag in vegetation response to radiation and temperature exceeded 6 months in certain areas of Amazon. Additionally, NDVI responses to shortwave radiation and temperature in the tropical rainforest ecosystem were opposite in different areas: positive in the north and negative in the south. This situation may have resulted from differences in the amounts of rainfall experienced in the two areas. Although precipitation is adequate across the tropical rainforest, there is a remarkable difference between the south and the north. In the northern region, there is almost no dry season (monthly rainfall less than 100 mm) [9]. A large amount of rain is accompanied by constant cloud cover, and consequently, vegetation greenness is radiation-limited; thus, enhanced shortwave radiation would result in more photosynthetic active radiation as well as strengthened vegetation photosynthesis [14].

We found that the relationships between precipitation and NDVI were negatively correlated during non-drought years in the Amazon rainforest. This means that a proper decrease in precipitation would not cause a decline in NDVI in non-drought years because the reduction of rain clouds would increase the shortwave radiation and thus promote the growth of vegetation in the tropical rainforest [14, 34]. Other research on the interaction of vegetation and climate has also revealed that an increase in precipitation can inhibit the greenness of vegetation in areas of the Amazon rainforest with sufficient rainfall [14, 22, 34]. Anderson et al. [2010] investigated the relationship between precipitation and vegetation using three indices (NDVI, EVI, NDWI), all of which showed a negative relation [14]. Even in dry periods, trees were able to use deep roots and hydrological redistribution to maintain water availability [47]. The observed negative relationship may also relate to the revealed dry-season vegetation greenness [1, 48, 49]. Nonetheless, in the 2005 and 2010 extreme droughts, sharply reduced precipitation still potentially contributed to the decline of NDVI. This may have occurred when precipitation decreased to a critical level of water required for vegetation growth. The impact of temperature on the growth of vegetation increased during the droughts. The decline of NDVI in the two extreme droughts resulted from the combined impacts of the three climate factors.

Some limitations and uncertainties existed in our study. The relationships between vegetation greenness and meteorological factors were assumed to be linear. In reality, the phenological mechanism of the tropical rainforest is complex [50], and the relationship between vegetation and climatic factors may not be completely linear. Our simplified approach was effective in exploring natural phenomenon and has been widely used [14, 22, 23, 51]. In such cases, further investigation beyond the general approach applied is needed. Moreover, there is a certain uncertainty in the response time of vegetation to climate change. Anderson et al. (2010) studied drought in the Amazon rainforest using remote sensing with the method of WCC and found that the vegetation index lag time in responding to climate elements was usually less than 6 months [14]. Wu et al. (2015) analyzed the time-lag effects of global vegetation responses to climate change. Moreover, they showed that the time lag of global vegetation response to temperature, precipitation and radiation was shorter than 3 months [22]. Sometimes the response of vegetation to droughts could be more than one year [38]. In this study, vegetation greenness responded to the three climatic factors with apparent time lags; most averaging interval periods associated with NDVI response to precipitation, radiation and temperature lasted 0–4, 0–9 and 0–6 months, respectively. Although the lag times were not the same, the general conclusion is similar: vegetation has a lagged response to climate. This paper differs from previous studies in that we considered that the influence of meteorological elements on vegetation is continuous and there exists a time interval. The interpretation of vegetation greenness was increased by this method, which indicates that the results of this paper are reasonable.

## 5. Conclusions

Overall, our results showed that the responses of vegetation greenness to precipitation, shortwave radiation and temperature differ markedly with respect to distinct regional vegetation types as well as water and heat conditions over the Amazon. Vegetation greenness responded to the three climatic factors with apparent and different lag periods. Evergreen broadleaf forests responded to precipitation with a time lag ranging from 0 to 2 months, which was shorter compared with savanna vegetation. The response of evergreen broadleaf forests and savanna to shortwave radiation indicates a longer time lag, and response to temperature varied spatially, with no concentration distribution characteristics.

Because of the complex phenological patterns of the Amazon, the overall ability of precipitation, radiation and temperature to explain variations in NDVI was low when time-lag effects were ignored. When time-lag effects were considered, their explanation ability significantly increased. In the tropical rainforest, these three factors explained 21.38% of NDVI variation with the time lag and increased relatively by 43.97%; in the savanna, they accounted for 75.40% of variation, a relative increase of 42.08%, and across the Amazon basin they accounted for 39.50% of the variation, a relative increase of 44.79%. These results suggest that considering lag effects gives us a better understanding of the interactions between Amazon vegetation and climatic factors.

Two kinds of indices (the explanation of each climate factor on NDVI and standardized regression coefficients between each climatic factor and NDVI) both demonstrated that vegetation greenness in most of the Amazon region was primarily affected by precipitation and radiation and was less affected by temperature in non-drought years. Nonetheless, in the common epicenter of the 2005 and 2010 droughts, the serious declines in Amazon vegetation were comprehensively induced by the rise in shortwave radiation and temperature and drop in precipitation. However, their contributions in the two droughts were not completely identical: in 2005, sharply enhanced shortwave radiation contributed more to the NDVI decline than in 2010; and in 2010, a remarkable rise in temperature made a greater contribution than in 2005. In our exploration, we found that there are many factors that cause vegetation greenness decrease. The research methods of this study can also be applied to other regions.

## Supporting information

S1 Fig Map of unchanged vegetation types in the Amazon region from 2001–2012 derived from MODIS land cover type data. EBF refers to evergreen broadleaf forest; DBF refers to deciduous broadleaf forest.
(DOCX)

S2 Fig Spatial distribution of dry-season NDVI anomalies from 2000–2014. (DOCX)

S1 Text Explanations of climatic factors on vegetation and the dominant climate factor. (DOCX)

S2 Text Variations in vegetation and climatic factors. (DOCX)
