# Supplementary material for: Climatic factors driving vegetation declines in the 2005 and 2010 Amazon droughts
Source: PLoS One. 2017 Apr 20;12(4):e0175379. doi: 10.1371/journal.pone.0175379 (PMC5398491; doi:10.1371/journal.pone.0175379)
Supplement: S1 Fig — EBF refers to evergreen broadleaf forest; DBF refers to deciduous broadleaf forest. (DOCX) [file pone.0175379.s001.docx]

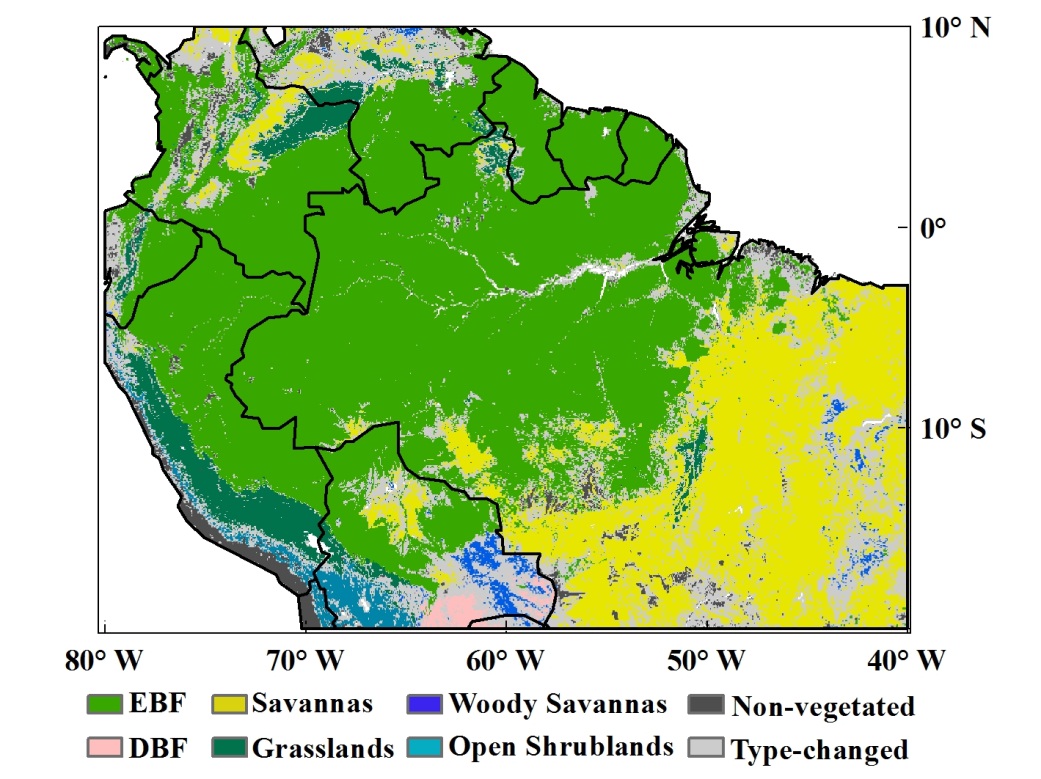


S1 Fig. Map of unchanged vegetation types in the Amazon region from 2001-2012 derived from MODIS land cover type data. EBF refers to evergreen broadleaf forest; DBF refers to deciduous broadleaf forest.
