# Supplementary figures and images for: Climatic factors driving vegetation declines in the 2005 and 2010 Amazon droughts
Source: PLoS One. 2017 Apr 20;12(4):e0175379. doi: 10.1371/journal.pone.0175379 (PMC5398491; doi:10.1371/journal.pone.0175379)

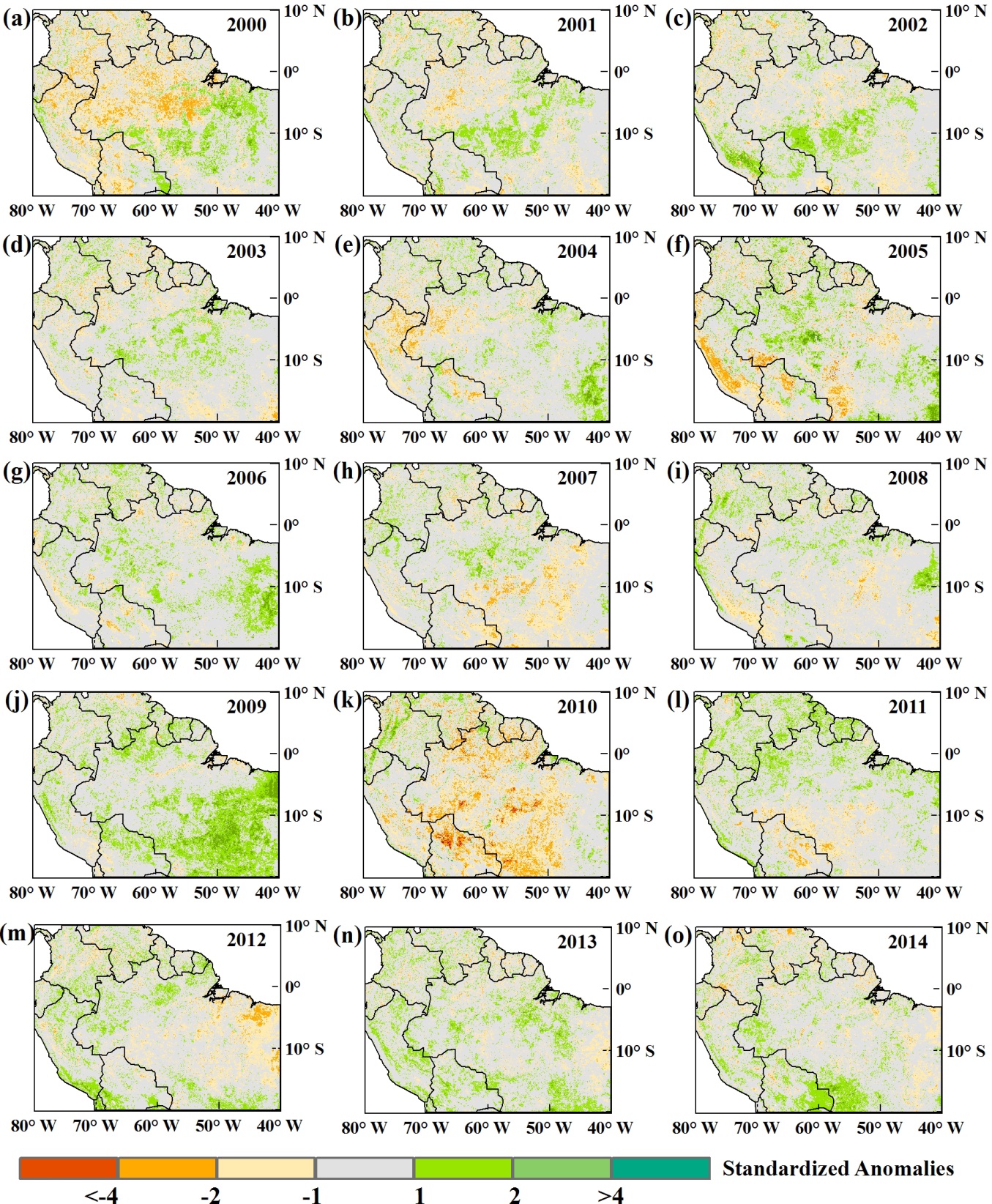


**S2 Fig.** Spatial distribution of dry-season NDVI anomalies from 2000-2014

Supplement: S2 Fig — (DOCX) [file pone.0175379.s002.docx]
