# Supplementary material for: Climatic factors driving vegetation declines in the 2005 and 2010 Amazon droughts
Source: PLoS One. 2017 Apr 20;12(4):e0175379. doi: 10.1371/journal.pone.0175379 (PMC5398491; doi:10.1371/journal.pone.0175379)
Supplement: S1 Text — (DOCX) [file pone.0175379.s003.docx]

## Explanations of climatic factors on vegetation and the dominant climate factor

The model is written as follows:

$$NDVI(i,j)=m_{1}*PRE\left( i,j \right)+ m_{2}*SWD\left( i,j \right)+m_{3}*TMP\left( i,j \right)+n\left( i,j \right) (1)$$

where *NDVI* is the monthly NDVI time series from 2000 to 2012, excluding 2005 and 2010; and *PRE*, *SWD* and *TMP* are time series with maximum correlations to NDVI, respectively. *m1*, *m_2_*, *m_3_* represent the regression coefficients of precipitation, shortwave radiation and temperature, respectively; *n* is the regression constant, and (i, j) is the pixel located in the row i and column j.

To explore the dominant climate factor governing vegetation greenness, we determined the relative contribution of each climatic variable. Two indices were adopted to indicate the contribution. One was the explanation (R^2^) of each climate factor on NDVI. Another was the standardized regression coefficient of each climate factor. In general, the independent variables involved in a multiple linear regression model differ by units of measurement as well as orders of magnitude. Therefore, the regression coefficients are not comparable and cannot accurately distinguish the impacts from different independent variables. To eliminate the effects caused by differences in measuring units and magnitude, we calculated standardized regression coefficients for the model. A standardized regression coefficient is usually used to determine the relative importance of each independent variable on the dependent variable [[46](#_ENREF_46)]. Standardized regression coefficients can be converted from non-standardized coefficients using the following formula:

*K _n_*^*^ = *k _n_*$\frac{S_{x_{n}}}{S_{y}}$ (2)

where *kn** is the standardized regression coefficient of a certain independent variable, *kn* is the non-standardized coefficient of the variable, and$S_{y}$ and $S_{x_{n}}$ are standard deviations of dependent variable *y* and independent variable *x_n_*, respectively.

Similarly, based on the unchanged vegetation type map, we conducted a statistical analysis to develop a comprehensive explanation of climate factors on different vegetation types and the contribution of each factor to that explanation.
