# Supplementary material for: Climatic factors driving vegetation declines in the 2005 and 2010 Amazon droughts
Source: PLoS One. 2017 Apr 20;12(4):e0175379. doi: 10.1371/journal.pone.0175379 (PMC5398491; doi:10.1371/journal.pone.0175379)
Supplement: S2 Text — (DOCX) [file pone.0175379.s004.docx]

## Variations in vegetation and climatic factors

The two droughts both occurred with the arrival of a dry season (July- September); Therefore, we first calculated the NDVI anomalies during the dry season to characterize the impact of the droughts on vegetation. The calculation was performed at the pixel scale using the following formula, and an identical reference period (i.e.2000-2014, excluding 2005 and 2010) was applied:

NDVI_yanomaly_*(i,j)*=$\frac{\mathrm{NDVI}y \left( i,j \right)-MEAN (\mathrm{NDVI}_{2000-2014}(i,j))}{STD (\mathrm{NDVI}_{2000-2014}(i,j))}$ (1)

where NDVIy_anomaly_(i, j) represents the dry-season NDVI anomaly of pixel (i, j) in 2005 (or 2010); NDVIy(i,j) represents the monthly average NDVI of pixel (i, j) in the 2005 (or 2010) dry season; *MEAN*(NDVI_2000-2014_(i,j)) represents the average dry-season NDVI from 2000 to 2014 excluding 2005 and 2010, which indicates the dry-season NDVI conditions in non-drought years; and *STD* represents the standard deviation of the dry-season NDVI in non-drought years. The anomaly value was normalized by division by the mean and indicates the amplitude of the variation relative to the mean.

Standardized anomalies for climatic factors were also computed. The anomalies indicated the extent of the departure from the average of non-drought years. Although many studies have evaluated climate anomalies by standardized approaches, most of them have ignored the time-lag effects and calculated the dry-season anomalies, simultaneously with NDVI. However, the correlation analysis of the NDVI and the three climatic factors demonstrated a phenomenon of vegetation greenness response to climate variables with a time lag. Therefore, we computed anomalies of the climatic factors during a period that affected dry-season (the most severe drought period) NDVI in the two droughts based on the lag effect.

Consider precipitation as an example to elaborate the method. The calculations of shortwave radiation and temperature anomalies follow a similar approach. For a specific pixel (i, j), first determine the time lag of NDVI response to precipitation. If the lag is 0 month, then calculate the precipitation anomalies of the dry season (July to September), then the same with that of NDVI. If the lag is 1 month, precipitation anomalies from June to September would be calculated; if the lag is two months, precipitation anomalies from May to September would be calculated, etc. The calculation formula is as follows, and an identical reference period (i.e.2000-2012, excluding 2005 and 2010) was applied:

PREy_anomaly_*(i,j)*=$\frac{\mathrm{PRE}y \left( i,j \right)-MEAN (\mathrm{PRE}_{2000-2012}(i,j))}{STD (\mathrm{PRE}_{2000-2012}(i,j))}$ (2)

where the PREy_anomaly_(i, j) is the 2005 (or 2010) precipitation anomaly; PREy(i, j) is 2005 (or 2010) monthly average precipitation of the specific months related to the time lag, which most influences the dry-season NDVI most, that is, the months corresponding to the optimum time lag obtained earlier; *MEAN* (PRE_2000-2012_(i, j)) is monthly average precipitation of the specific months in non-drought years; and *STD* is the standard deviation corresponding to the mean.
